# Impact of airway dysfunction on dental health

**DOI:** 10.6026/97320630016026

**Published:** 2020-01-15

**Authors:** Juliette Tamkin

**Affiliations:** 1 Dental Group of Sherman Oaks, California, CA 1403, USA

## Abstract

The airway encompasses the mouth, jaw, nasal passages, tongue, and throat. Dentists in collaboration with other health professionals can recognize the early signs of the disorder, perform proper assessment of facial and dental structures, and provide treatment to help prevent and manage the dysfunction. Therefore, it is of interest to discuss the impact of airway development and dysfunction on dental health.

## Background:

Clinical rationale:

Breathing is an important human function. The healthiest and most effective way to breathe is through the nose. Nasal breathing aids in the proper development of the upper airway and associated structures (i.e. skeletal and dental anatomical growth). Mouth breathing, caused by an obstruction of the upper airway can result in, enlarged tonsils and adenoids, bruxism causing wear and fracture of teeth, temporo-mandibular disorder of the jaw joints, myofascial pain, erosion of the teeth, malocclusion, periodontal disease, caries and impacted teeth. Beyond the dental implications, upper airway obstructions can lead to sleep disturbed breathing which causes headaches, snoring, difficulty sleeping, neck, jaw, or ear pain. Chronic diseases such as obesity, ADHD, asthma, anxiety, Alzheimer’s, type II diabetes, cardiovascular disease and sleep apnea develop from sleep disturbed breathing and thus affect the quality of life and life expectancy.

## Clinical approach:

The first step in identifying patients with an airway dysfunction is to use an airway questionnaire and an in-office physical examination that includes photographs. During the examination there should be an evaluation of facial form and symmetry, an exam of the nose, the tonsils and adenoids, the tongue, the teeth, and the soft and hard palates. A home sleep study using a cardiopulmonary coupling device should be completed for a patient suspected to have airway dysfunction based on the questionnaire and examination. A follow-up with a formal sleep study in a lab with a sleep MD may need to be performed to achieve a complete and accurate diagnosis for a patient suspected to have apnea.

## Clinical assessment:

Proper formation of oral structures consists of a wide U-shaped maxilla and mandible, no malocclusion, space for the tongue to rest properly at the anterior roof of the mouth, and a wide enough nose to create open nasal passages that promote nasal breathing. Poor facial development and asymmetry are two of the most easily identified signs of airway dysfunction. Bottle-feeding, weaning to soft foods, thumb sucking, pacifier use, and mouth breathing are the main causes of poor facial development. The function of the nose is to take in air, which is then warmed, moistened, and filtered. Small amounts of nitric oxide, which play a role in killing dust mites and helps reduce inflammation, is added to the air before going into the lungs. Mouth breathing unfortunately provides none of these benefits. The healthcare provider should evaluate for septal deviations, size of the inferior turbinates and nasal valve stenosis during the evaluation of the nose. Referral to an allergist, ENT, and myofunctional therapist may be needed to achieve proper nasal breathing.

Pediatric mouth breathing is reported in 10-15% of children [1]. Characteristic features of mouth breathers is the "Long-faced Syndrome" which describes a long face appearance, dropped eyes, dark spots under eyes, open lips, narrow nostrils, weak cheek muscles, high palate, narrowing of the upper jaw and malocclusion. Mouth breathing patients may report of symptoms such as, dry lips and mouth, snoring and open mouth while sleeping, chronic sinus and ear infections and colds, chronic bad breath, and swollen and red gums that bleed easily. Malocclusions that can be seen associated with airway dysfunction and accompanying poor facial development are open bites, cross bites, impacted teeth, and tooth crowding. Mouth breathers and patients with tongue and lip ties also exhibit signs of altered posture of the tongue, speech deficits, and a swallow abnormality.

Mouth breathing directly affects dental health by causing the drying of oral structures and the decrease of saliva production. Saliva acts to neutralize acid in the mouth and helps to flush away bacteria. Without saliva and its beneficial protective mechanisms, risk of decay and periodontal disease, the pathological inflammation of the gum and bone support surrounding the teeth, increases [2]. During sleep, mouth breathing decreases intra oral pH as compared to normal breathing [3]. This lowered pH can lead to erosion of tooth surfaces, increased sensitivity of the teeth to temperatures and susceptibility to tooth decay.

Ankyloglossia, also known as a tongue tie, is an embryological remnant of tissue in the midline between the under surface of the tongue and the floor of the mouth that may restrict normal tongue movement. A restricted tongue will exhibit limited proper movement during speech and swallowing. It can make it difficult for infants to properly nurse and thrive. For children, lip and tongue ties can hinder a child’s ability to maintain good oral hygiene. Lip and tongue ties create a food trap between the soft tissues of the mouth and the teeth, preventing proper cleaning and movement of food off of tooth surfaces. The extended time food remains on the teeth increases the risk of caries. Gum recession, the loss of gingival height and volume around teeth, can be caused by the constant tension from the ties on the gingiva surrounding teeth. Recession of gingiva exposes the root surfaces of teeth to the intra-oral environment. This may cause tooth sensitivity and an increased risk of root erosion and caries.

Bruxism is an oral para-functional activity of teeth grinding and/or jaw clenching. As muscles of the body relax during sleep, the tethered tongue, resting low in the mouth, can fall backward and obstruct the airway, causing difficulty in breathing. The brain responds by sending signals to the jaw to slide forward or protrude, thus opening the airway to allow air into the body. Unfortunately, this protrusive sliding of the lower jaw against the upper jaw causes abrasive grinding of tooth surfaces. Airway related bruxism can lead to loss of tooth structure known as abfractions, cracks in teeth, mobility of teeth, bone loss, pain, and early tooth loss.

Tonsils and Adenoids are another set of critical structures in the airway paradigm. They are lymphatic tissues on each side of the back of the throat and at the junction of where the nasal passages meet the throat. These tissues function to filter out viruses and bacteria and produce antibodies to fight off infection. By age 4, the tonsils and adenoids are fully developed and their size can be graded on a 1-4 scale. Mouth Breathing increases turbulence of breath going directly into the throat and allergies causing post-nasal drip of mucous result in chronic inflammation of these two structures. Enlarged tonsils block airways making it difficult to breath, while also creating or making worse sleep disturbed breathing. If airway restriction exists from these tissues, it is recommended they be removed. Adults only require tonsil removal since the Adenoids receed as we age.

Snoring and mouth breathing are two primary indicators of sleep disturbed breathing. Obstructive sleep apnea affects approximately 20% of US Adults, of whom about 90% are un-diagnosed [4]. During sleep disturbed breathing, the brain unconsciously recognizes it is not getting enough oxygen and will signal a state of alert, the fight-or-flight response. Our body adrenal glands respond by releasing stress hormone and adrenaline. Over time the adrenaline causes the body's immune system to create chronic inflammation. Sleep is no longer a time for rest, repair, and regeneration, a critical compromise for optimum health. For children, the chronic activation of the sympathetic nervous system, coupled with the lack of oxygen, excess carbon dioxide and fragmented sleep, can lead to ADHD, learning disabilities, anxiety, depression, and aggressive behaviour [5]. During snoring, apnea, and mouth breathing, children’s brains and bodies cannot develop correctly. One of the neurological deficits of poor sleep quality, is lower IQ in children [6]. There is approximately a 2.5-fold increase in upper airway resistance during sleep while mouth breathing as compared to nasal breathing in normal subjects. When using the Apnea-Hypopnea Index, obstructive apneas and Hypopneas are more frequent when breathing orally (AHI 43) than nasally (AHI 1.5) [7]. Other factors contribute to obstructive sleep apnea, such as obesity, tobacco use, and alcohol consumption, but the basic problem is structural. If we can improve a child’s airway development we can thus prevent the structural compromises that are the core cause of sleep disturbed breathing and its related comorbidities.

## Conclusion:

As a standard of care, an airway questionnaire and physical examination should be completed on all new and existing patients. If an airway dysfunction is suspected, the patient should be made aware of the risk and benefits of treatment. Referrals to an airway centric dentist, airway centric orthodontist, ENT, myo functional therapist, oral surgeon, allergist, and sleep MD should be completed. It is imperative that healthcare professionals use a strong team-based approach to manage all aspects of the patients with under developed airways. In-depth education on airway dysfunction should be implemented at the graduate school level and in continuing education courses for all healthcare professionals to promote collaborative treatment. Thus, with education, early intervention, and collaboration we can help patients to live long with a healthy life.

## About the author:

Juliette Tamkin is a dentist in Sherman Oaks, California. She provides comprehensive dental care with a focus on improving overall health and wellness.

## References

[1] Denotti G (2014). Journal of Pediatric and Neonatal Individualized Medicine.

[2] Frenkel ES, Ribbeck K (2015). Applied and Environmental Microbiology.

[3] Choi JE (2016). Journal of Oral Rehabilitation.

[4] Finkel KJ (2009). Sleep Med.

[5] Bonuck K (2012). Pediatrics.

[6] Kaemingk KL (2003). Journal of the International Neuropsychological Society.

[7] Fitzpatrick MF (2003). Eur Respir J..

